# Seasonal variations in hospital admissions and case-fatality of ischemic stroke: a nationwide analysis of >4.2 million cases in Germany

**DOI:** 10.3389/fepid.2026.1750089

**Published:** 2026-04-30

**Authors:** Omar Hahad, Simon-Noah Hakim-Meibodi, Seyed Hamed Rastguye Haghi, Sasan Faridi, Andreas Daiber, Alexandra Schneider, Kathrin Wolf, Nikolaos Nikolaou, Volker H. Schmitt, Philipp Lurz, Christine Espinola-Klein, Yafang Cheng, Andrea Pozzer, Jos Lelieveld, Thomas Münzel, Daniel Wollschläger, Lukas Hobohm, Karsten Keller

**Affiliations:** 1 Department of Cardiology, University Medical Center of the Johannes Gutenberg-University Mainz, Mainz, Germany; 2 German Center for Cardiovascular Research (DZHK), Partner Site Rhine Main, Mainz, Germany; 3 Center for Air Pollution Research (CAPR), Institute for Environmental Research, Tehran University of Medical Sciences, Tehran, Iran; 4 Institute of Epidemiology, Helmholtz Zentrum München GmbH, German Research Center for Environmental Health, Neuherberg, Germany; 5 Center for Thrombosis and Hemostasis (CTH), University Medical Center of the Johannes Gutenberg-University Mainz, Mainz, Germany; 6 Max Planck Institute for Chemistry, Mainz, Germany; 7 Climate and Atmosphere Research Center, The Cyprus Institute, Nicosia, Cyprus; 8 Institute of Medical Biostatistics, Epidemiology and Informatics (IMBEI), University Medical Center of the Johannes Gutenberg-University Mainz, Mainz, Germany

**Keywords:** ischemic stroke, season, summer, winter, case-fatality, hospital admission

## Abstract

**Background:**

Ischemic stroke is a leading cause of global morbidity and mortality, with seasonal variations potentially influencing both outcomes. While previous studies have suggested a pronounced association of the cold months with increased stroke morbidity and mortality, the evidence remains limited and inconsistent. This study aimed to assess seasonal variations in ischemic stroke hospital admissions and in-hospital case-fatality and complications in Germany over an 18-year period.

**Methods:**

This nationwide retrospective analysis included all hospitalizations for ischemic stroke in Germany from 2005 to 2022, using data from the Federal Statistical Office. Patients were categorized by season of hospital admission (winter, spring, summer, autumn). Multivariable logistic regression models were used to assess the association between season and in-hospital case-fatality, adjusting for age, sex, and comorbidities.

**Results:**

A total of 4,236,789 ischemic stroke hospitalizations were analyzed. No statistically significant seasonal variation in stroke hospitalization was observed. However, in-hospital case-fatality was significantly higher in winter (7.4%) compared to summer (6.6%, *p* < 0.001). This seasonal association was independent of patient age, sex, and comorbidities [adjusted odds ratio (OR): 1.140, 95% confidence interval (CI): 1.128–1.152; *p* < 0.001]. Similar trends were observed in both men (adjusted OR: 1.122, 95% CI: 1.103–1.141; *p* < 0.001) and women (adjusted OR: 1.112, 95% CI: 1.096–1.128; *p* < 0.001), without substantial sex-specific differences.

**Conclusion:**

While ischemic stroke hospital admissions remained stable across seasons, in-hospital case-fatality was significantly increased during winter compared to summer. These findings highlight the need for targeted seasonal prevention and management strategies. Further research is needed to explore underlying mechanisms and evaluate potential interventions to mitigate excess winter case-fatality among stroke patients.

## Graphical Abstract

**Figure:**
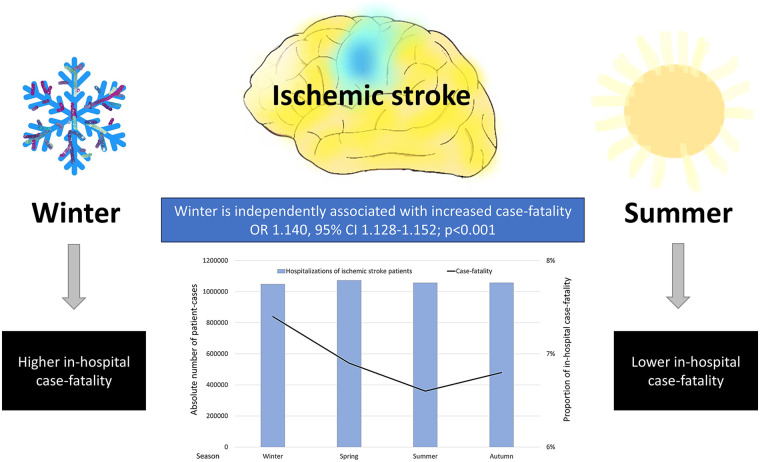

## Introduction

Ischemic stroke remains a major cause of global mortality and morbidity worldwide, with substantial implications for public health and healthcare systems (1). In addition to well-established risk factors such as arterial hypertension (2), diabetes mellitus (3), atrial fibrillation (4), and dyslipidemia (5), there is growing evidence suggesting that environmental factors, including ambient temperature and also other exposures with seasonal variations, may also influence risk of ischemic stroke and related complications (6).

According to the latest data from the Global Burden of Disease (GBD) study, ischemic stroke was the fourth leading cause of death worldwide (Level 4) for the year 2021, accounting for over 3.59 million deaths [confidence interval (CI): 3.21–3.89 million]. Non-optimal temperatures, including low (220,024 deaths; CI: 187,894–255,960) and high (36,228 deaths; CI: 2,273–86,556) temperatures, contributed to a total of 254,878 (CI: 196,804–330,865) ischemic stroke-related deaths (7).

A recent meta-analysis of 20 epidemiological studies showed an increased stroke morbidity and mortality, with hot ambient temperature associated with a 10% increase in stroke morbidity [relative risk (RR): 1.10, 95% CI: 1.02–1.18] and a 9% increase in stroke mortality (RR: 1.09, 95% CI: 1.02–1.17), while cold ambient temperature was linked to a 33% increase in stroke morbidity (RR: 1.33, 95% CI: 1.17–1.51) and an 18% increase in stroke mortality (RR: 1.18, 95% CI: 1.06–1.31) (8). Winter-related cardiovascular disease may be driven by multiple mechanisms, including increased blood pressure due to cold-induced vasoconstriction, impaired endothelial function promoting atherosclerosis, altered lipid metabolism and diet changes with higher intake of saturated fats, reduced physical activity leading to weight gain and metabolic dysregulation, air pollution exposure triggering systemic inflammation and oxidative stress, heightened coagulation and thrombogenesis increasing the risk of thrombotic events, and psychosocial stressors such as cold stress, all of which may collectively contribute to an increased stroke morbidity and mortality in winter (9).

Several studies have investigated the relationship between stroke incidence and mortality and seasonal variations, but the findings have been inconsistent. These differences in the study results are partly due to variations in the study populations (such as community-based vs. hospital-based cohorts) and regional climate conditions (9–11). Additionally, large-scale/nationwide research on how seasonal changes affect hospital admissions, case-fatality, and in-hospital complications of stroke is limited, even though such information could inform the development of national seasonal prevention and management strategies. In a large prospective stroke cohort (*N* = 569,307) analyzing stroke seasonality, the results showed a winter excess in in-hospital mortality (+10.3%) and prolonged hospital stays (+7.3%). Winter admissions were linked to higher odds of in-hospital mortality [odds ratio (OR): 1.023, 95% CI: 1.006–1.040]. Stroke-related complications like pneumonia (+6.7%) and sepsis (+3.2%) also showed a winter excess (12).

The present study aims to make a substantial contribution to the existing body of evidence by examining seasonal variations in ischemic stroke hospital admissions and in-hospital case-fatality and complications in Germany. This will be achieved through the analysis of a large, nationwide dataset covering nearly two decades and including more than 4.2 million stroke hospital admissions.

## Methods

### Data source

For the present study, the German nationwide inpatient statistics was analysed assessing all hospitalizations due to ischemic stroke within the years 2005–2022 (source: Research Data Center of the Federal Statistical Office and the Statistical Offices of the federal states, DRG Statistics 2005–2022, and own calculations). Patients' diagnoses are coded according to ICD-10-GM (International Classification of Diseases, 10th Revision with German Modification) and diagnostical, surgical and interventional procedures are coded with the OPS codes [surgery, diagnostic and procedures codes (Operationen- und Prozedurenschlüssel)]. The Federal Statistical Office of Germany (Statistisches Bundesamt, Wiesbaden, Germany) gathers all treatment data from nearly all inpatient cases of Germany [processed according to the diagnosis related groups (DRG) system] (13, 14).

In the present study, all hospitalized patients with a primary diagnosis of ischemic stroke (ICD I63), meaning all patients admitted due to ischemic stroke between 2005 and 2022, were included. The primary diagnosis refers to the condition that is chiefly responsible for the patients’ hospitalization (15). The study analyses were performed on our behalf by the RDC of the Federal Statistical Office and the Statistical Offices of the federal states in Wiesbaden (Germany). The aggregated statistics were provided on basis of SPSS codes (SPSS® software, version 20.0, SPSS Inc., Chicago, Illinois), which were supplied from us to the RDC. Monthly mean air temperature in Germany was obtained by the German meteorological service (Deutscher Wetterdienst).

### Diagnoses, procedural codes, and definitions

In Germany, diagnoses are coded according to the International Classification of Diseases and Related Health Problems, 10th Revision, with German Modification (ICD-10-GM). Diagnostic, surgical, and interventional procedures are coded using the German Procedure Classification (OPS—Operationen- und Prozedurenschlüssel).

Hospitalizations of ischemic stroke patients were stratified by season, focusing on patients with the highest and lowest case-fatality rates. Consequently, hospitalizations of patients admitted in winter (highest case-fatality) and summer (lowest case-fatality) were compared. The seasons were defined as follows: winter (December–February), spring (March–May), summer (June–August), and fall (September–November). As aforementioned, monthly mean air temperature (in °C) and average precipitation (in mm) data for Germany were obtained from the German Meteorological Service (Deutscher Wetterdienst).

The Charlson Comorbidity Index is the most extensively studied tool for quantifying comorbidity burden (16, 17). The calculation of the Charlson Comorbidity Index was done according the established definitions and was reported in detail previously (17).

### Study outcome

The primary outcome of the study was in-hospital case-fatality, defined as all-cause mortality occurring during the hospital stay. Sex-specific analyses were performed to assess whether seasonal differences in in-hospital case-fatality varied between men and women. Additionally, we analyzed the absolute number of hospitalizations due to ischemic stroke during the different seasons.

### Ethical aspects

Since this study did not involve direct access to individual patient data by us as investigators, approval from an ethics committee and informed consent were not required, in accordance with German law.

### Statistical methods

The seasonally stratified data were compared regarding patients' characteristics as well as relevant outcomes of hospitalizations. Continuous variables were tested using the Mann–Whitney *U* test, and categorical variables were analyzed with the chi-square test or Fisher's exact test, as appropriate. Additionally, the absolute numbers of ischemic stroke hospitalizations across different seasons were compared using the Kruskal–Wallis test. Given the very large sample size, even small absolute differences in proportions can reach statistical significance. Therefore, *p*-values were interpreted alongside absolute differences and clinical relevance rather than on the basis of statistical significance alone.

Univariable and multivariable logistic regression models were used to investigate the association of season on in-hospital case-fatality [winter vs. summer season (as reference)]. Results are presented as OR with corresponding 95% CI. The multivariable logistic regression model was used to assess whether the season (winter vs. summer) independently predicted in-hospital case-fatality. The model was adjusted for the following factors: I) age, sex, cancer, heart failure, essential arterial hypertension, chronic obstructive pulmonary disease (COPD), acute and/or chronic kidney failure, obesity, diabetes mellitus, hyperlipidemia, peripheral artery disease, and coronary artery disease; or II) the Charlson comorbidity index. We provided these logistic regression analyses in the subgroups of male and female patients as well as regardless of patients’ sex. Additionally, monthly and seasonal trends in the absolute numbers of ischemic stroke hospitalizations and case fatality were illustrated. In addition, we calculated linear regression models to analyze the association between i) monthly average air temperature as well as ii) average precipitation as exposures and a) hospitalizations due to ischemic stroke as well as b) in-hospital case-fatality rate as outcomes. The software SPSS® (versions 20.0 and 25.0; SPSS Inc., Chicago, Illinois) was used for the computerized analysis. *P*-values < 0.05 (two-sided) were considered statistically significant. *P*-values are not adjusted for multiple testing.

## Results

### Seasonal distribution of ischemic stroke hospital admissions

**Figure 1:**
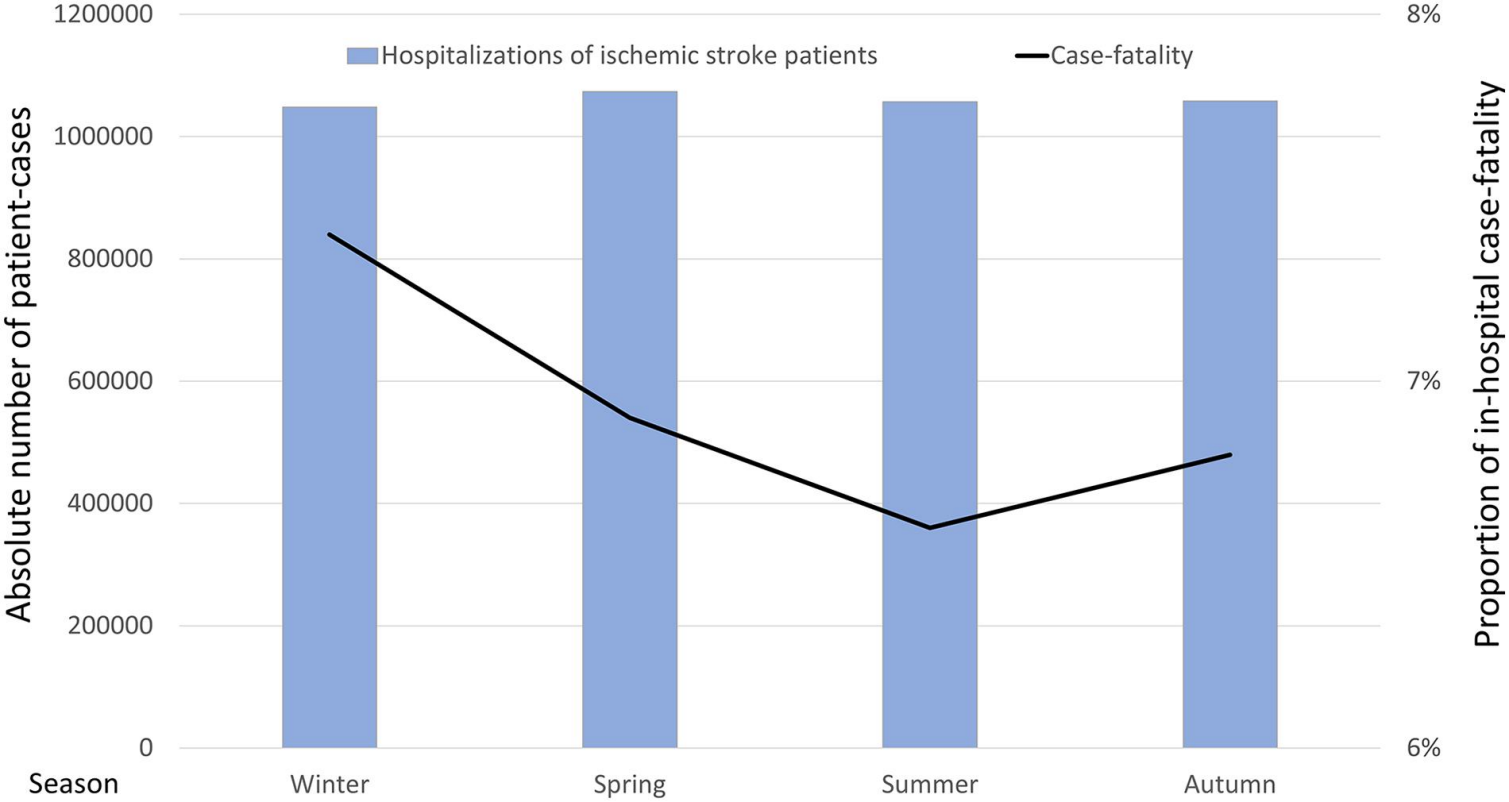
Absolute number of hospitalizations due to ischemic stroke in Germany (2005–2022) (blue bars, left *y*-axis) and associated in-hospital case-fatality (black line, right *y*-axis), stratified by season.

**Figure 2:**
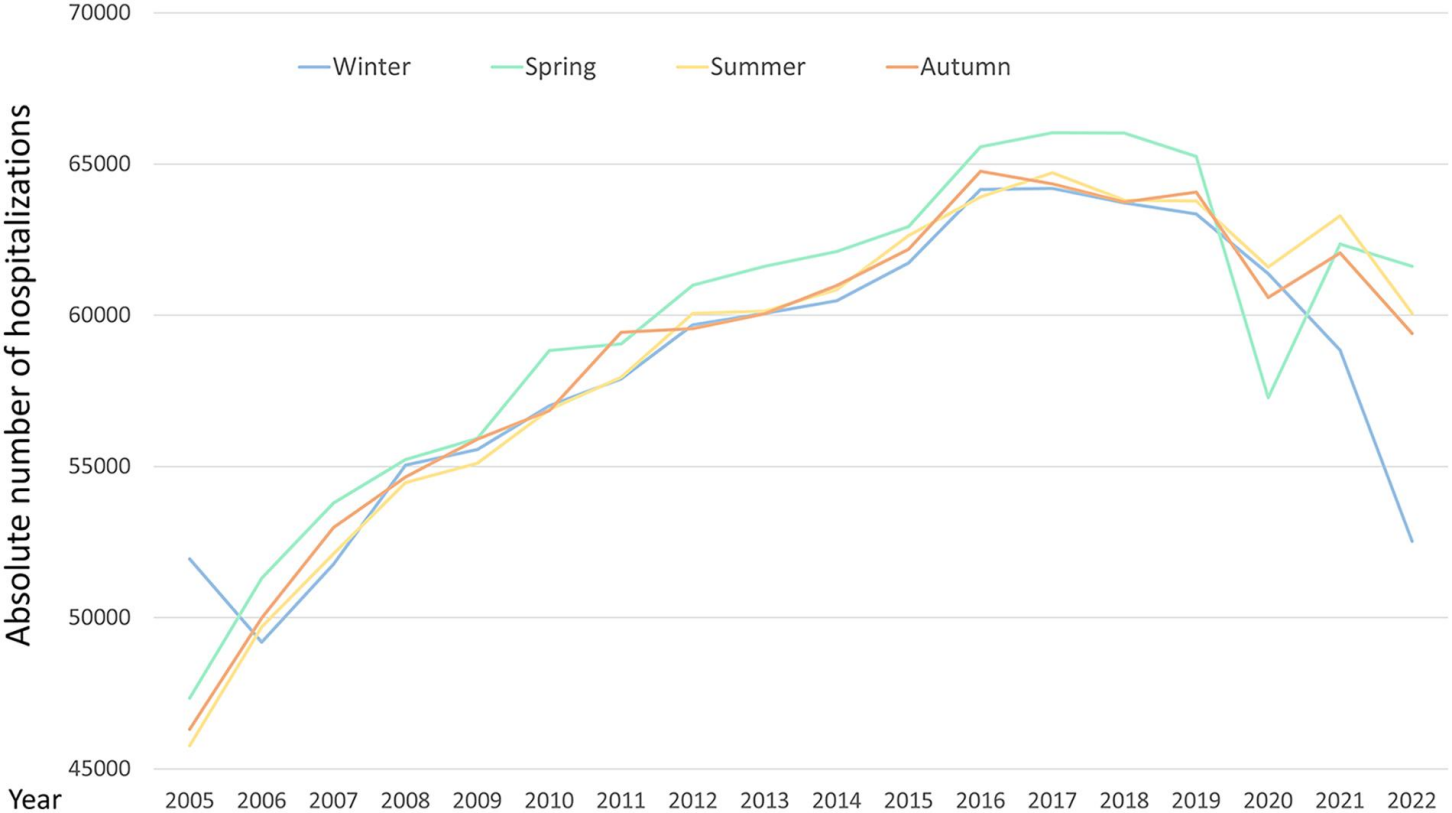
Absolute number of hospitalizations due to ischemic stroke in Germany across seasons for each year from 2005 to 2022.

A total of 4,236,789 hospitalizations for ischemic stroke in Germany between 2005 and 2022 were included in this nationwide analysis. Ischemic stroke patients had a median age of 76 years [interquartile range (IQR): 66.0–83.0] with a slight male predominance (Table 1). Notably, the prevalence of atrial fibrillation/flutter among hospitalizations for ischemic stroke was higher in winter (29.5%) compared to summer (27.9%, *p* < 0.001). Adverse in-hospital complications such as pneumonia (5.2% vs. 4.4%; *p* < 0.001) and myocardial infarction (1.4% vs. 1.3%; *p* = 0.001) were more common in winter than in summer (Table 1). The absolute number of hospital admissions caused by ischemic stroke remained stable across seasons with no significant seasonal variation observed (winter: 24.7%, spring: 25.3%, summer: 24.9%, fall: 25.0%; *p* = 0.631) (Table 1, Figure 1). Additionally, we calculated a *T*-Test with 95%CI: Mean difference between summer and winter was −153.22, 95% CI: −881.30 to 574.86; *p* = 0.677. This consistency was further supported by annual stratification, which demonstrated no major fluctuations in the seasonal distribution of admissions over the 18-year study period (Figure 2).

**Table 1:** Patient characteristics of 4,236,789 ischemic stroke hospitalizations stratified by season.

Variable	Winter	Spring	Summer	Fall	*P* for comparison between winter and summer
Mean air temperature ( °C)	1.69	8.96	17.83	9.86	
Mean precipitation (mm)	62.42	54.36	80.09	57.94	
Seasonal (3-month) hospitalizations due to ischemic stroke per 100,000 citizens	70.86	72.53	71.42	71.49	0.635
Number of hospitalizations due to ischemic stroke events	1,048,603 (24.7%)	1,073,334 (25.3%)	1,056,877 (24.9%)	1,057,975 (25.0%)	0.631
Length of in-hospital stay (days)	9.0 (5.0–15.0)	9.0 (6.0–15.0)	9.0 (5.0–14.0)	9.0 (5.0–15.0)	<0.001
Median age (years)	76.0 (66.0–83.0)	76.0 (66.0–83.0)	76.0 (66.0–83.0)	76.0 (66.0–83.0)	<0.001
Age >70 years	718,695 (68.5%)	730,713 (68.1%)	710,560 (67.2%)	720,803 (68.1%)	<0.001
Female sex*	521,230 (49.7%)	529,454 (49.3%)	515,242 (48.8%)	523,378 (49.5%)	<0.001
Obesity	54,049 (5.2%)	57,223 (5.3%)	56,973 (5.4%)	54,033 (5.1%)	<0.001
Diabetes mellitus	299,697 (28.6%)	309,995 (28.9%)	305,430 (28.9%)	301,797 (28.5%)	<0.001
Hyperlipidemia	388,770 (37.1%)	401,261 (37.4%)	401,035 (37.9%)	401,318 (37.9%)	<0.001
Essential arterial hypertension	704,536 (67.2%)	725,240 (67.6%)	711,518 (67.3%)	713,881 (67.5%)	<0.001
Coronary artery disease	155,715 (14.8%)	161,087 (15.0%)	160,026 (15.1%)	158,896 (15.0%)	<0.001
Heart failure	116,547 (11.1%)	119,889 (11.2%)	116,366 (11.0%)	116,657 (11.0%)	0.027
Atrial fibrillation/flutter	308,936 (29.5%)	309,743 (28.9%)	294,820 (27.9%)	306,475 (29.0%)	<0.001
Cancer	29,911 (2.9%)	30,510 (2.8%)	31,305 (3.0%)	30,873 (2.9%)	<0.001
Chronic obstructive pulmonary disease	48,795 (4.7%)	49,917 (4.7%)	49,303 (4.7%)	48,464 (4.6%)	0.099
Peripheral artery disease	35,937 (3.4%)	37,334 (3.5%)	37,713 (3.6%)	36,886 (3.5%)	<0.001
Acute and/or chronic kidney failure	154,204 (14.7%)	158,109 (14.7%)	157,377 (14.9%)	157,638 (14.9%)	0.001
Charlson comorbidity index	6.00 (5.00–8.00)	6.00 (5.00–8.00)	6.00 (5.00–8.00)	6.00 (5.00–8.00)	<0.001
Treatments					
Chronic oral anticoagulation therapy	124,272 (11.9%)	126,404 (11.8%)	124,922 (11.8%)	128,400 (12.1%)	<0.001
Catheter-directed treatment for ischemic stroke	34,925 (3.3%)	35,423 (3.3%)	35,429 (3.4%)	36,925 (3.5%)	<0.001
Lysis therapy for ischemic stroke	109,964 (10.5%)	113,300 (10.6%)	112,395 (10.6%)	113,626 (10.7%)	<0.001
Adverse in-hospital complications					
Myocardial Infarction	14,260 (1.4%)	14,404 (1.3%)	13,766 (1.3%)	14,047 (1.3%)	0.001
Pneumonia	54,385 (5.2%)	50,898 (4.7%)	46,208 (4.4%)	48,603 (4.6%)	<0.001
Deep vein thrombosis and/or thrombophlebitis	8,993 (0.9%)	9,016 (0.8%)	9,455 (0.9%)	9,432 (0.9%)	0.008
Pulmonary embolism	5,180 (0.5%)	4,965 (0.5%)	5,084 (0.5%)	5,036 (0.5%)	0.010
Shock	4,154 (0.4%)	4,164 (0.4%)	4,036 (0.4%)	4,015 (0.4%)	0.171
Bleeding with requirement of transfusion of blood constituents	20,480 (2.0%)	20,734 (1.9%)	20,727 (2.0%)	20,888 (2.0%)	0.870
In-hospital case-fatality	77,520 (7.4%)	74,516 (6.9%)	69,245 (6.6%)	72,026 (6.8%)	<0.001

* Data on sex available for *N* = 4,236,653 hospitalized ischemic stroke patients.

### Seasonal variations in in-hospital case-fatality

**Figure 3:**
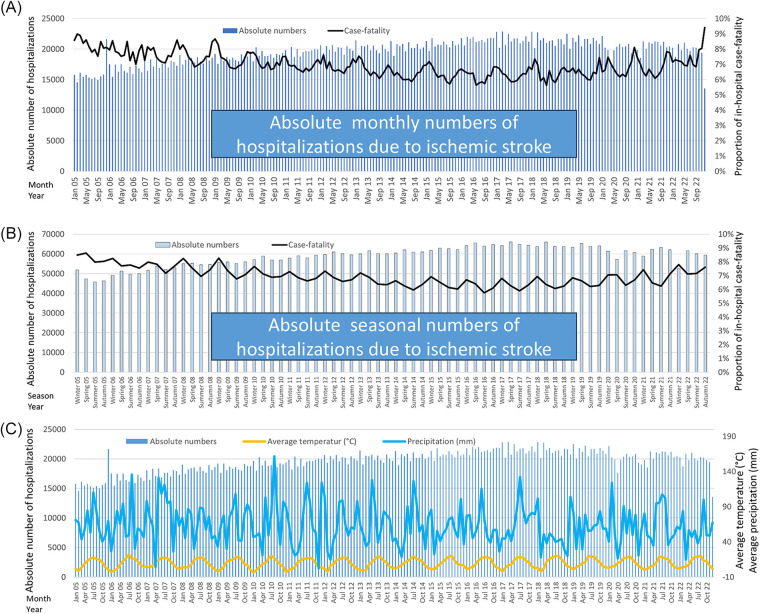
Absolute number of hospitalizations due to ischemic stroke (blue bars, left *y*-axis) and in-hospital case fatality (black line, right *y*-axis) in Germany, stratified by month **(A)** and season **(B)**, 2005–2022. In panel (**C**), the absolute monthly numbers of hospitalizations due to ischemic stroke (blue bars, left *y*-axis) and average monthly air temperature (yellow line, right *y*-axis) as well as average monthly precipitation (light blue line, right *y*-axis) in Germany were illustrated for the observational period 2005–2022.

In contrast to admissions, in-hospital case-fatality exhibited a pronounced seasonal pattern with the highest case-fatality occurring in winter (7.4%) and the lowest in summer (6.6%) (*p* < 0.001) (Table 1, Figure 3). Monthly analysis revealed a peak in-hospital case-fatality during December and January, with the lowest case-fatality observed in July and August (Figure 3). This trend remained consistent across the study period, suggesting a robust association between colder months and increased in-hospital case-fatality.

### Regression analysis of seasonal in-hospital case-fatality

Multivariable logistic regression analyses confirmed that winter admissions were independently associated with increased in-hospital case-fatality compared to summer (adjusted OR: 1.116, 95% CI: 1.104–1.129; *p* < 0.001), even after adjusting for age, sex, and comorbidities (Table 2). This association persisted when adjusting for the Charlson comorbidity index (adjusted OR: 1.140, 95% CI: 1.128–1.152; *p* < 0.001). Subgroup analyses revealed no substantial sex-specific differences, with similarly elevated odds of case-fatality in both men (adjusted OR: 1.122, 95% CI: 1.103–1.141; *p* < 0.001) and women (adjusted OR: 1.112, 95% CI: 1.096–1.128; *p* < 0.001) during winter. The results remained stable after additional adjustment for catheter-directed treatment for ischemic stroke and lysis therapy for ischemic stroke (Table 2).

**Table 2:** Comparison of in-hospital case-fatality between winter and summer seasons.

Overall	Univariable Odds ratio (95% CI)	*P*-value	Multivariable* Odds ratio (95% CI)	*P*-value	Multivariable^†^ Odds ratio (95% CI)	*P*-value	Multivariable^‡^ Odds ratio (95% CI)	*P*-value
In-hospital case-fatality	1.139 (1.127–1.151)	<0.001	1.116 (1.104–1.129)	<0.001	1.140 (1.128–1.152)	<0.001	1.031 (1.026–1.035)	<0.001
Men								
In-hospital case-fatality	1.141 (1.123–1.160)	<0.001	1.122 (1.103–1.141)	<0.001	1.141 (1.122–1.160)	<0.001	1.028 (1.021–1.036)	<0.001
Women								
In-hospital case-fatality	1.130 (1.114–1.145)	<0.001	1.112 (1.096–1.128)	<0.001	1.135 (1.119–1.151)	<0.001	1.032 (1.025–1.038)	<0.001

* Adjusted for: age, sex, cancer, heart failure, arterial hypertension, chronic obstructive pulmonary disease, acute and/or chronic kidney failure, obesity, diabetes mellitus, hyperlipidemia, peripheral artery, disease, and coronary artery disease.

† Adjusted for: Charlson comorbidity index.

^‡^ Adjusted for: age, sex, cancer, heart failure, arterial hypertension, chronic obstructive pulmonary disease, acute and/or chronic kidney failure, obesity, diabetes mellitus, hyperlipidemia, peripheral artery, disease, coronary artery disease, catheter-directed treatment for ischemic stroke and lysis therapy for ischemic stroke.

In this context, the results of our analyses confirmed that pulmonary embolism (adjusted OR: 4.558, 95% CI: 4.405–4.716; *p* < 0.001) as well as bleeding events with requirement of transfusion of blood constituents (adjusted OR: 3.141, 95% CI: 3.085–3.199; *p* < 0.001) were important triggers of in-hospital case-fatality in patients with ischemic stroke, but, notably, only pulmonary embolism and pneumonia showed a significant variation, whereas myocardial infarction and bleeding events with requirement of transfusion of blood constituents did not (Table 3).

**Table 3:** Comparison of other outcomes than survival between winter and summer seasons.

Overall	Univariable Odds ratio (95% CI)	*P*-value	Multivariable* Odds ratio (95% CI)	*P*-value	Multivariable^†^ Odds ratio (95% CI)	*P*-value	Multivariable^‡^ Odds ratio (95% CI)	*P*-value
Myocardial infarction	1.006 (0.996–1.016)	0.216	1.007 (0.997–1.017)	0.173	1.006 (0.996–1.016)	0.239	1.007 (0.997–1.018)	0.153
Pneumonia	1.047 (1.041–1.052)	<0.001	1.047 (1.041–1.052)	<0.001	1.048 (1.042–1.054)	<0.001	1.047 (1.042–1.053)	<0.001
Pulmonary embolism	1.026 (1.009–1.043)	0.002	1.025 (1.008–1.042)	0.003	1.141 (1.122–1.160)	<0.001	1.026 (1.009–1.043)	0.003
Bleeding with requirement of transfusion of blood constituents	1.000 (0.992–1.009)	0.916	1.000 (0.992–1.008)	0.996	1.001 (0.992–1.009)	0.873	1.001 (0.992–1.009)	0.878

* Adjusted for: age, sex, cancer, heart failure, arterial hypertension, chronic obstructive pulmonary disease, acute and/or chronic kidney failure, obesity, diabetes mellitus, hyperlipidemia, peripheral artery, disease, and coronary artery disease.

^†^ Adjusted for: Charlson comorbidity index, catheter-directed treatment for ischemic stroke and lysis therapy for ischemic stroke.

^‡^ Adjusted for: age, sex, cancer, heart failure, arterial hypertension, chronic obstructive pulmonary disease, acute and/or chronic kidney failure, obesity, diabetes mellitus, hyperlipidemia, peripheral artery, disease, coronary artery disease, catheter-directed treatment for ischemic stroke and lysis therapy for ischemic stroke.

When computing the relation between temperature and precipitation on the one hand and stroke hospitalizations and in-hospital case-fatality on the other hand, average monthly air temperature [beta_per 1 °C change_: 7.821 (95% CI: −30.317 to 45.959), *P* = 0.686] as well as monthly average precipitation [beta_per 1 mm change_: −1.492 (95% CI: −10.348 to 7.365), *P* = 0.740] were both not associated with monthly numbers of stroke hospitalizations. In contrast, lower average monthly air temperature [beta_per 1 °C change_: −9.757 (95% CI: −11.441 to −8.073), *P* < 0.001] as well as lower monthly average precipitation [beta_per 1 mm change_: −0.588 (95% CI: −1.077 to −0.098), *P* = 0.019] were significantly associated with increased in-hospital case-fatality. This analysis should support the findings of the seasonal results.

## Discussion

The present nationwide analysis of more than 4.2 million hospitalizations of patients admitted due to ischemic stroke in Germany between 2005 and 2022 provides compelling evidence for significant seasonal variation in in-hospital case-fatality with a pronounced increase during winter compared to summer, while stroke admission rates remained stable across seasons. These findings contribute important new insights to the growing body of literature on seasonal patterns in stroke outcomes.

Several previous studies have reported an increased stroke mortality during winter months, which aligns with our observation of higher in-hospital case-fatality in the cold season and cold temperatures. For instance, a study in Canada using administrative health data and examining 394,145 stroke/TIA hospitalizations revealed critical seasonal patterns with winter showing the most pronounced effects (18). The 30-day case fatality risk was highest in winter at 12.4%, compared to 11.4% in summer (HR: 1.10, 95% CI: 1.07–1.13). Interestingly, the authors found that winter showed significantly lower stroke hospitalizations than summer (RR: 0.94, 95% CI: 0.94–0.95), suggesting that seasonal variation may differentially influence stroke incidence vs. survival. This observation is in line with our findings, which highlight pronounced and distinct associations of winter with case-fatality, while hospitalizations were not affected by seasonal variations. Similarly, the STROMA study of 7,129 first-ever stroke cases found no seasonal variation in overall stroke incidence (19). In contrast, case-fatality risks peaked significantly during winter (15.1% overall, 17.2% in women vs. 12.5% in men). Further supporting this, the analysis of 1,039 stroke patients demonstrated that those admitted in spring and winter presented with higher baseline stroke severity and experienced significantly worse 90-day functional outcomes compared to other seasons (20). In accordance, a population-based study of Northern Portugal indicated that the relative risk of a fatal vs. a non-fatal stroke increased by 15.5% (95% CI: 6.1–25.4%) for a 1 °C lower maximum temperature over the previous day (21). Polcaro-Pichet and colleagues reported that the results of their study suggest that cold temperature and snowfall are independent risk factors for death from hemorrhagic stroke in male patients (22). Overall, these heterogeneous findings underline that the direction and magnitude of seasonal effects on stroke outcomes likely depend on a combination of climatic conditions, healthcare system characteristics, and study design, rather than season *per se*.

However, the nationwide analysis of 1,422,496 stroke hospitalizations in Brazil revealed peak hospitalization rates during winter months (median temperature 23.8 °C) (23). In contrast, the mean winter temperature in our German dataset was 1.69 °C (Table 1), indicating that “winter” in Brazil reflects a much milder climatic context with substantially less cold-related physiological stress. This profound difference in background winter temperature is likely a key driver of the divergent seasonal patterns and limits direct comparability between the two settings, in addition to differences in healthcare systems and study designs. Furthermore, a Chinese study showed that ischemic stroke incidence was highest in autumn (OR: 1.22, 95% CI: 1.06–1.40, compared with spring), again underscoring that seasonal patterns may vary across regions depending on local climate, population characteristics, and healthcare structures (24).

The observed excess winter case-fatality of ischemic stroke is likely multifactorial, reflecting both biological and systemic drivers. The observed pattern of increased winter case-fatality without seasonal variation in admissions likely reflects distinct pathophysiological and systemic mechanisms affecting post-stroke outcomes rather than incidence (18). Cold-induced vasoconstriction and hemoconcentration elevate blood pressure, impair collateral perfusion, and promote thrombosis, thereby worsening infarct severity (25, 26). Concomitantly, winter peaks of respiratory infections exacerbate systemic inflammation and immune dysregulation, predisposing stroke patients to pneumonia and sepsis, complications (27–29) that were significantly more frequent in our dataset. Thus, hospital systems factors further compound this risk: resource strain from seasonal respiratory illnesses may delay critical interventions, while prolonged immobilization increases pneumonia and thromboembolic events (30, 31). In particular, pulmonary embolism as well as bleeding events with requirement of transfusion of blood constituents are independent triggers of in-hospital case-fatality in patients with ischemic stroke, but only pulmonary embolism and pneumonia showed a significant seasonal variation.

The higher prevalence of atrial fibrillation and heart failure among winter admissions further amplifies cardiovascular vulnerability, increasing the risk of myocardial infarction or fatal arrhythmias (13, 32, 33). Study identified infections, heart failure and atrial fibrillation as important drivers of increased in mortality in hospitalized patients, underlining the importance of comorbidity burden and in particular the occurrence of infections and atrial fibrillation for short term survival in hospitalized patients (34–36). Beyond biology, health system strain during winter and potential delays in hospital presentation may compromise timely reperfusion therapy (13), while increased immobilization contributes to thromboembolic and pulmonary complications (37). Taken together, these interlinked mechanisms provide a pathophysiological rationale for the observed seasonal excess in in-hospital case-fatality, despite stable admission rates across seasons. This dissociation of unchanged seasonal stroke incidence, but increased in-case fatality of stroke in the winter underscores that winter mortality may thus comprise primarily drivers of post-stroke pathophysiological cascades rather than incidence enhancers (e.g., atherosclerosis).

The strengths of our study comprise its nationwide coverage, which eliminates the selection biases inherent in single-center studies, and the use of standardized ICD-10-GM and OPS coding across all participating hospitals, ensuring diagnostic consistency. The 18-year study period captures multiple complete climate cycles and allows observation of long-term trends of in-hospital outcomes.

These findings have important implications for clinical practice and public health. Hospitals may need to enhance monitoring for winter-specific complications such as infections and thromboembolic events in stroke patients. Preventive strategies could include intensified risk factor management as winter approaches, particularly for high-risk patients. At the public health level, awareness campaigns could help educate the population about winter-specific stroke risks and the importance of prompt care-seeking even during holiday periods.

## Limitations

There are certain limitations to our study that require consideration: I) The study results are based on ICD and OPS discharge codes, which may lead to incomplete data due to under-reporting/under-coding. II) Important clinical data, such as information about disability status or concomitant medications, are missing. III) One major limitation of our study is that the cause of death is not available in the dataset of the German nationwide inpatient statistics. IV) Data about medications were only very limited available and data about arrival time/onset-to-door time were not available in the German nationwide inpatient statistics. V) The data only cover the in-hospital course, and data regarding later follow-up are not available in the German nationwide inpatient statistics, meaning that we may underestimate the full seasonal effect if winter strokes lead to increased post-discharge mortality that we could not capture. Additionally, while Germanys temperate climate makes our findings highly relevant for similar regions, they may not fully generalize to areas with more extreme seasonal temperature variations and other climate regions. Despite these limitations, we observed a clear and strong seasonal variation of short-term mortality in patients hospitalized due to ischemic stroke in all of the annual analyses. The focus of our study was on the well-defined and reliable endpoints of hospital admission and in-hospital case-fatality.

## Conclusions

In conclusion, this large nationwide observational study describes a consistent seasonal pattern in short-term in-hospital case-fatality of ischemic stroke with higher mortality during winter months despite stable admission rates. These findings generate population-level hypotheses about potential biological and healthcare-system mechanisms underlying excess winter mortality. Future studies with detailed clinical, treatment, and severity data are needed to identify modifiable causal pathways and to determine whether seasonally targeted prevention and management strategies could help reduce winter case-fatality among stroke patients.

## Data Availability

The data analyzed in this study is subject to the following licenses/restrictions: Not permissible. Requests to access these datasets should be directed to post@destatis.de.

## References

[1] Li X Kong X Yang C Cheng Z Lv J Guo H Global, regional, and national burden of ischemic stroke, 1990–2021: an analysis of data from the global burden of disease study 2021. EClinicalMedicine. (2024) 75:102758. 10.1016/j.eclinm.2024.102758 39157811 PMC11327951

[2] McManus M Liebeskind DS. Blood pressure in acute ischemic stroke. J Clin Neurol. (2016) 12(2):137–46. 10.3988/jcn.2016.12.2.137 26833984 PMC4828558

[3] Chen R Ovbiagele B Feng W. Diabetes and stroke: epidemiology, pathophysiology, pharmaceuticals and outcomes. Am J Med Sci. (2016) 351(4):380–6. 10.1016/j.amjms.2016.01.011 27079344 PMC5298897

[4] Migdady I Russman A Buletko AB. Atrial fibrillation and ischemic stroke: a clinical review. Semin Neurol. (2021) 41(4):348–64. 10.1055/s-0041-1726332 33851396

[5] Tziomalos K Athyros V Karagiannis A Mikhailidis D. Dyslipidemia as a risk factor for ischemic stroke. Curr Top Med Chem. (2009) 9(14):1291–7. 10.2174/156802609789869628 19849661

[6] Ranta A Ozturk S Wasay M Giroud M Béjot Y Reis J. Environmental factors and stroke: risk and prevention. J Neurol Sci. (2023) 454:120860. 10.1016/j.jns.2023.120860 37944211

[7] Hahad O Frenis K Kuntic M Daiber A Münzel T. Accelerated aging and age-related diseases (CVD and neurological) due to air pollution and traffic noise exposure. Int J Mol Sci. (2021) 22(5):2419. 10.3390/ijms22052419 33670865 PMC7957813

[8] Wen J Zou L Jiang Z Li Y Tao J Liu Y Association between ambient temperature and risk of stroke morbidity and mortality: a systematic review and meta-analysis. Brain Behav. (2023) 13(7):e3078. 10.1002/brb3.3078 37269140 PMC10338745

[9] Fares A. Winter cardiovascular diseases phenomenon. N Am J Med Sci. (2013) 5(4):266–79. 10.4103/1947-2714.110430 23724401 PMC3662093

[10] Li Y Zhou Z Chen N He L Zhou M. Seasonal variation in the occurrence of ischemic stroke: a meta-analysis. Environ Geochem Health. (2019) 41(5):2113–30. 10.1007/s10653-019-00265-y 30848411

[11] Kuzmenko NV Galagudza MM. Dependence of seasonal dynamics of hemorrhagic and ischemic strokes on the climate of a region: a meta-analysis. Int J Stroke. (2022) 17(2):226–35. 10.1177/17474930211006296 33724111

[12] Lorking N Wood AD Tiamkao S Clark AB Kongbunkiat K Bettencourt-Silva JH Seasonality of stroke: winter admissions and mortality excess. Clin Neurol Neurosurg. (2020) 199:106261. 10.1016/j.clineuro.2020.106261 33096427

[13] Keller K Hobohm L Münzel T Ostad MA. Sex-specific differences regarding seasonal variations of incidence and mortality in patients with myocardial infarction in Germany. Int J Cardiol. (2019) 287:132–8. 10.1016/j.ijcard.2019.04.035 31005418

[14] Keller K Hobohm L Ebner M Kresoja KP Munzel T Konstantinides SV Trends in thrombolytic treatment and outcomes of acute pulmonary embolism in Germany. Eur Heart J. (2020) 41(4):522–9. 10.1093/eurheartj/ehz236 31102407

[15] Deutsche Krankenhausgesellschaft (DKG), GKV-Spitzenverband, Verband der privaten Krankenversicherung (PKV) und Institut für das Entgeltsystem im Krankenhaus (InEK GmbH). *Deutsche Kodierrichtlinien Version 2018*. Berlin: InEK GmbH. (2017). https://www.g-drg.de/media/files/archiv/drg-systemjahr-2018-datenjahr-2016/kodierrichtlinien/deutsche-kodierrichtlinien-2018-druckversion-a4-pdf

[16] Charlson ME Pompei P Ales KL MacKenzie CR. A new method of classifying prognostic comorbidity in longitudinal studies: development and validation. J Chronic Dis. (1987) 40(5):373–83. 10.1016/0021-9681(87)90171-8 3558716

[17] Keller K Schmitt VH Hahad O Espinola-Klein C Munzel T Lurz P Categorization of patients with pulmonary embolism by Charlson comorbidity Index. Am J Med. (2024) 137(8):727–35. 10.1016/j.amjmed.2024.04.025 38663792

[18] Brissette V Kapral MK Yu B Fang J Odugbemi T Shamy M Seasonal variations in stroke occurrence. Neuroepidemiology. (2025) 59:236–45. 10.1159/000540056 39008950

[19] Khan FA Engstrom G Jerntorp I Pessah-Rasmussen H Janzon L. Seasonal patterns of incidence and case fatality of stroke in Malmö, Sweden: the STROMA study. Neuroepidemiology. (2005) 24(1-2):26–31. 10.1159/000081046 15459506

[20] Liu Y Gong P Wang M Zhou J. Seasonal variation of admission severity and outcomes in ischemic stroke—a consecutive hospital-based stroke registry. Chronobiol Int. (2018) 35(3):295–302. 10.1080/07420528.2017.1369430 29372813

[21] Magalhães R Silva MC Correia M Bailey T. Are stroke occurrence and outcome related to weather parameters? Results from a population-based study in northern Portugal. Cerebrovasc Dis. (2011) 32(6):542–51. 10.1159/000331473 22104569

[22] Polcaro-Pichet S Kosatsky T Potter BJ Bilodeau-Bertrand M Auger N. Effects of cold temperature and snowfall on stroke mortality: a case-crossover analysis. Environ Int. (2019) 126:89–95. 10.1016/j.envint.2019.02.031 30784804

[23] Kurtz P Bastos LSL Aguilar S Hamacher S Bozza FA. Effect of seasonal and temperature variation on hospitalizations for stroke over a 10-year period in Brazil. Int J Stroke. (2021) 16(4):406–10. 10.1177/1747493020947333 32752950

[24] Jin H Xu Z Li Y Xu J Shan H Feng X Seasonal variation of stroke incidence in Wujin, a city in southeast China. Health Sci Rep. (2018) 1(4):e29. 10.1002/hsr2.29 30623065 PMC6266434

[25] Chen X Shang W Huang X Shu L Xiao S Jiang Q The effect of winter temperature on patients with ischemic stroke. Med Sci Monit. (2019) 25:3839–45. 10.12659/MSM.916472 31120864 PMC6556065

[26] Teixeira CE Webb RC. Cold-induced vasoconstriction. Circ Res. (2004) 94(10):1273–5. 10.1161/01.RES.0000131755.49084.04 15166113

[27] Paganini-Hill A Lozano E Fischberg G Perez Barreto M Rajamani K Ameriso SF Infection and risk of ischemic stroke. Stroke. (2003) 34(2):452–7. 10.1161/01.STR.0000053451.28410.98 12574559

[28] Lieberman D Lieberman D Porath A. Seasonal variation in community-acquired pneumonia. Eur Respir J. (1996) 9(12):2630–4. 10.1183/09031936.96.09122630 8980980

[29] Danai PA Sinha S Moss M Haber MJ Martin GS. Seasonal variation in the epidemiology of sepsis*. Crit Care Med. (2007) 35(2):410–5. 10.1097/01.CCM.0000253405.17038.43 17167351

[30] Nakajima M Watanabe-Hara R Inatomi Y Hashimoto Y Uchino M. Respiratory infectious complications after acute ischemic stroke. Rinsho Shinkeigaku. (2002) 42(10):917–21. https://pubmed.ncbi.nlm.nih.gov/12739378/12739378

[31] O'Brien EC Wu J Zhao X Schulte PJ Fonarow GC Hernandez AF Healthcare resource availability, quality of care, and acute ischemic stroke outcomes. J Am Heart Assoc. (2017) 6(2):e003813. 10.1161/JAHA.116.003813 28159820 PMC5523738

[32] Russo V Solimene F Zanotto G Pisano EC Della Bella P Iacopino S Seasonal trend of ventricular arrhythmias in a nationwide remote monitoring database of implantable defibrillators and cardiac resynchronization devices. Int J Cardiol. (2019) 275:104–6. 10.1016/j.ijcard.2018.10.025 30327133

[33] Spencer FA Goldberg RJ Becker RC Gore JM. Seasonal distribution of acute myocardial infarction in the second national registry of myocardial infarction. J Am Coll Cardiol. (1998) 31(6):1226–33. 10.1016/S0735-1097(98)00098-9 9581712

[34] Farooqui AA Mazhar Uddin SM Maheshwari SK Clements K Ashraf R Keith J New onset atrial fibrillation in hospitalized patients. J Community Hosp Intern Med Perspect. (2025) 15(2):25–32. 10.55729/2000-9666.1467 40309291 PMC12039332

[35] Shaver CM Chen W Janz DR May AK Darbar D Bernard GR Atrial fibrillation is an independent predictor of mortality in critically ill patients*. Crit Care Med. (2015) 43(10):2104–11. 10.1097/CCM.0000000000001166 26154932 PMC4725582

[36] Lee E Choi EK Han KD Lee H Choe WS Lee SR Mortality and causes of death in patients with atrial fibrillation: a nationwide population-based study. PLoS One. (2018) 13(12):e0209687. 10.1371/journal.pone.0209687 30586468 PMC6306259

[37] Keller K Hobohm L Munzel T Konstantinides SV Lankeit M. Sex-specific and age-related seasonal variations regarding incidence and in-hospital mortality of pulmonary embolism in Germany. ERJ Open Res. (2020) 6(2):00181-2020. 10.1183/23120541.00181-2020 32607372 PMC7306502

